# Creutzfeldt-Jakob Disease Following Kidney Transplantation

**DOI:** 10.7759/cureus.21632

**Published:** 2022-01-26

**Authors:** Neeraj Sharma, Lin Wang, Hima Namboodiri

**Affiliations:** 1 University of Southern California Transplant, Department of Nephrology, University of Southern California, Los Angeles, USA; 2 Division of Nephrology and Hypertension, University of Southern California, Los Angeles, USA; 3 Internal Medicine, Keck School of Medicine, Los Angeles, USA

**Keywords:** kidney transplantation, cns, opportunistic infections, creutzfeldt-jakob disease, prion disease, acute encephalopathy

## Abstract

Neurologic complications after kidney transplantation are frequent and related to immunosuppressive therapy and vintage dialysis time, including comorbidities such as diabetes, hypertension, and cardiovascular diseases. Creutzfeldt-Jakob disease (CJD) is a very rare and rapidly fatal neurodegenerative disorder. Kidney transplant recipients present with mixed and complex neuropsychiatric manifestations, which makes diagnosis challenging. This case highlights the importance of enhancing awareness of the clinical presentation and timely diagnosis of this rare, fatal neurodegenerative disorder.

## Introduction

Kidney transplant recipients are at high risk of developing neurologic complications. Approximately, one-third of transplant recipients experience neurologic alterations [1]. Creutzfeldt-Jakob disease (CJD) is an infrequent, rapidly progressive, fatal neurodegenerative disease caused by mutated prion proteins. In humans, prion diseases are very rare, with an annual incidence rate of approximately one to two people per million, and can be classified as sporadic, acquired, or familial [2]. It is often difficult and challenging to diagnose CJD in the transplant population. Common clinical manifestations of CJD include rapid and progressive cognitive decline, cerebellar dysfunction, myoclonus, and personality and behavioral changes. All types of CJD are progressive and transmissible. The disease course follows a rapid decline of cognitive and functional impairment toward akinetic mutism and eventually death, often within one year of disease onset [3].

## Case presentation

Here, we present a case of a 71-year-old woman with a history of end-stage kidney disease due to presumed diabetic nephropathy who underwent a deceased donor kidney transplant with thymoglobulin induction in November 2018. She achieved excellent allograft function and was maintained on triple immunosuppressive therapy with tacrolimus, mycophenolate mofetil, and prednisone. Her post-transplant course was unremarkable, and she remained in good health up until two years after transplantation, when she developed encephalopathy with psychotic features, including generalized weakness and falls.

The family reported that the patient had problems getting dressed and was also getting lost in her own home. A week prior to admission, the family disclosed that the patient had started experiencing visual hallucinations and having persecutory delusions of paranoia.

Her symptoms were initially thought to be due to an adverse effect of tacrolimus, for which she was switched to sirolimus. Blood work included a complete metabolic profile, complete blood counts, thyroid studies, human immunodeficiency virus, folate, thiamine, and vitamin B12, all of which were normal. Heavy metal work-up, including mercury, lead, arsenic, zinc, copper, and ceruloplasmin, were within normal limits. Computerized tomography of the chest, abdomen, and pelvis showed no signs of malignancy; paraneoplastic markers in serum were negative; urine toxicology was negative.

Non-contrast magnetic resonance imaging (MRI) of the brain showed nonspecific basal ganglia enhancement and was not consistent with a stroke or posterior reversible encephalopathy syndrome. An electroencephalogram (EEG) showed normal recordings in wakefulness and sleep. A repeat MRI with contrast was notable for symmetric signal abnormalities involving the caudate and putamen and, to a lesser extent, the bilateral thalami as well as the involvement of the cortex.

Based on clinical symptoms and imaging findings, the possibility of prion disease was considered. Lumbar puncture was performed with normal opening pressures and cell counts. Cerebrospinal fluid (CSF) glucose was slightly elevated at 89 mg/dl (normal 40-70 mg/dl). Protein in the CSF was normal. Paraneoplastic autoantibodies in the CSF were negative. PCRs for herpes simplex virus 1 and 2, human herpesvirus 1, 2, 6, enterovirus, adenovirus, parvovirus, cytomegalovirus, varicella-zoster virus, Epstein-Barr virus, acid-fast bacilli, mycoplasma pneumonia, toxoplasma, and cryptococcal antigen were all negative. The 14-3-3 protein was detected in the CSF, including an elevated Tau protein of 2281 pg/ml (normal 0-1149 pg/ml). Real-time quaking-induced conversion (RT-QuIC) was also positive. The patient’s mental status remained unchanged throughout her hospitalization. Given the lack of effective treatment for prion diseases, the patient was treated conservatively. Ultimately, the patient was discharged to a skilled nursing facility with plans to be transitioned to palliative care.

## Discussion

Creutzfeldt-Jakob disease is a transmissible spongiform encephalopathy in which the pathologic particle is the prion, a proteinaceous infectious particle that lacks nucleic acid [4]. The accumulation of abnormal prion proteins leads to neuronal degeneration, astrocytic gliosis, and spongiform changes, resulting in a uniformly fatal neurologic disease. Prion disease has been reported in the general population. One case of CJD was reported after liver transplantation in 1995 [5]. However, after an extensive review of published reports within kidney transplant recipients, we found no reports of CJD.

As mentioned earlier, there are three categories of CJD: sporadic, acquired, and familial. The most common type, sporadic CJD (sCJD), occurs in about 85% of cases. A recent review of the global incidence of CJD found that cases of sCJD compared to other etiologies of CJD may be increasing, potentially because of an aging population [6]. Familial CJD (fCJD) is seen in patients with a family history of CJD or who test positive for a genetic mutation in the prion protein gene PRNP, and they account for about 5-10% of all CJD cases. Iatrogenic CJD (iCJD), which occurs via contaminated neurosurgical instruments and following corneal or dural graft transplantation, accounts for less than 1% of all CJD cases. A variant form (vCJD) is a recognized form of CJD but has not been classified in any major category. It is thought to be transmitted by ingestion of beef infected with bovine spongiform encephalopathy. The vCJD was first identified in the United Kingdom (UK), in 1996 [6]. Molesworth et al. investigated 177 cases in the UK of vCJD and found one situation where a liver transplant recipient developed vCJD post-transplantation [7]. 

**Figure 1:**
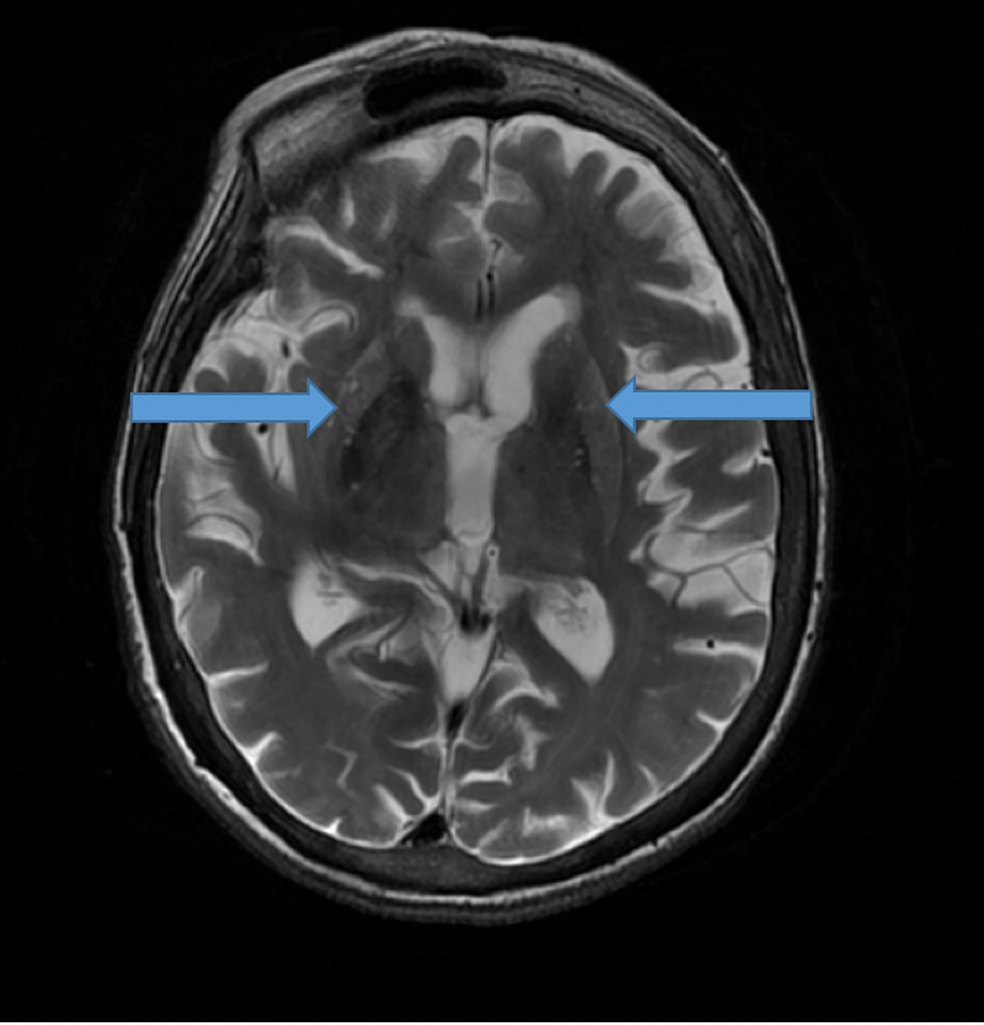
T2-weighted magnetic resonance image showing bilateral symmetric signal abnormality involving the caudate and putamen (blue arrows).

CJD is often a diagnostic dilemma for medical providers, especially in the solid organ transplant population where neurologic diseases can be due to neurodegenerative, toxic, metabolic, infectious, neoplastic, or autoimmune etiologies. Therefore, an extensive workup for reversible and treatable causes is needed. The diagnosis of definite sCJD requires either an autopsy or a brain biopsy, though these are rarely performed [8]. The diagnoses of probable sCJD, fCJD, and iCJD require fulfillment of multiple criteria, including clinical and familial context, cerebrospinal fluid markers, and imaging modalities [9]. The diagnosis of CJD has improved in recent years with the adoption of MRI as the imaging of choice (Figure 1) and the development of specific CSF analysis.

There is no definitive treatment for CJD. The mainstay of treatment is symptomatic and supportive care. A few drug trials were performed in CJD patients; unfortunately, none of them demonstrated a clear survival benefit [10-12]. Despite advances in understanding the mechanism of this disease, the prognosis is extremely poor.

## Conclusions

The diagnosis of new neuropsychiatric symptoms in solid-organ transplant recipients has a wide differential. This case demonstrates a rare diagnosis of CJD in a kidney transplant patient, which has not been reported previously. Because CJD is always fatal, an early and accurate diagnosis is critical so that family and care providers can understand the prognosis and develop a management plan.
